# Endoparasites of European hedgehogs (*Erinaceus europaeus*) in Germany and their zoonotic potential: proposed *Capillaria ovoreticulata* genetically identified as *Capillaria putorii*

**DOI:** 10.1186/s13071-025-06858-0

**Published:** 2025-06-07

**Authors:** Karolin Schütte, Andrea Springer, Florian Brandes, Maximilian Reuschel, Michael Fehr, Angela Kern, Christina Strube

**Affiliations:** 1 Institute for Parasitology, Centre for Infection Medicine, University of Veterinary Medicine Hannover, Hanover, Germany; 2 Wildlife Rescue and Conservation Center Sachsenhagen, Sachsenhagen, Germany; 3 Department of Small Mammal, Reptile and Avian Diseases, University of Veterinary Medicine Hannover, Hanover, Germany; 4 Megacor Diagnostik GmbH, Hoerbranz, Austria

**Keywords:** *Crenosoma striatum*, Lungworms, *Giardia*, *Cryptosporidium*, Helminths, Nematodes, Trematodes, Cestodes, Gastrointestinal parasites, Protozoa

## Abstract

**Background:**

European hedgehogs (*Erinaceus europaeus*) are frequently infected with a variety of endoparasites. The hedgehogs’ synanthropic lifestyle results in frequent contact with pets and humans, posing the risk of parasite spillover from a One Health perspective.

**Methods:**

The present study assessed the endoparasite fauna and excretion intensity of 531 European hedgehogs presented at wildlife rehabilitation centres in Germany. Faecal samples were examined by the combined sedimentation–flotation method, the Baermann technique and *FAST*est® CRYPTO-GIARDIA Strips (MEGACOR Diagnostik GmbH) from July 2018 to May 2021. *Cryptosporidium* spp. and *Giardia* spp. positive samples were further differentiated via amplification of the 60 kDa glycoprotein gene and the β-giardin gene, respectively. In addition, molecular identification of adult intestinal *Capillaria* spp. and Acanthocephala spp. was achieved via the mitochondrial cytochrome c oxidase subunit 1 (*cox-1*) gene.

**Results:**

Endoparasite prevalence was 95.5% (507/531). The most frequently detected helminth species was *Crenosoma striatum* (77.6%, [412/531]), followed by *Capillaria erinacei* (68.2%, [362/531]), *Capillaria putorii* (68.2%, [362/531]), *Capillaria aerophila* (26.7%, [142/531]), *Brachylaemus erinacei* (5.1%, [27/531]), undetermined trematode eggs (0.2% [1/531]) and *Hymenolepis nana* (0.2%, [1/531]). Detected protozoans included coccidia (12.8%, [68/531]), *Cryptosporidium* spp. (11.9%, [63/531]) and *Giardia* spp. (1.3%, [7/531]). Acanthocephala spp. were present in 1.5% (8/531) of samples, and two examined specimens were molecularly identified as *Plagiorhynchus cylindraceus*. Infections with *C. aerophila* showed a significant seasonal pattern and a negative correlation with bodyweight. For the remaining parasites, no significant associations with age, bodyweight, survival or seasonality were observed. Molecular typing revealed the presence of *Cryptosporidium parvum* subtype IIa prevalence of 2.1%, [11/531]), IIc (0.9%, [5/531]) and IId (0.6%, [3/531]), *Cryptosporidium erinacei* subtype XIIIa (6.1%, [33/531]) and XIIIb (0.2%, [1/531]), and *Giardia duodenalis* (sub)assemblage A(1) (1.3%, [7/531]).

**Conclusions:**

The hedgehogs showed high infection rates with pulmonary and gastrointestinal helminths. Molecular analysis clarified the species distribution of the gastrointestinal *Capillaria*, consisting of *C. erinacei* and *C. putorii*, disproving the existence of the previously described *Capillaria ovoreticulata*. Furthermore, molecular typing of *Cryptosporidium* and *Giardia* spp. revealed zoonotic subtypes and (sub)assemblages. In addition, *C. aerophila* and *H. nana* may infect humans. Therefore, precautionary measures should be taken when handling hedgehogs to mitigate the zoonotic risk.

**Graphical Abstract:**

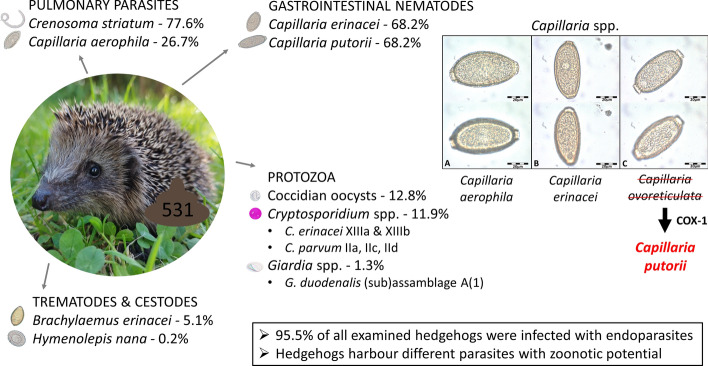

**Supplementary Information:**

The online version contains supplementary material available at 10.1186/s13071-025-06858-0.

## Background

The European hedgehog (*Erinaceus europaeus*) belongs to the family Erinaceidae, the oldest group of mammals, whose members were already present in the Cretaceous period [1]. This shows that hedgehogs are highly adaptable to habitat changes [2], but a population decline has been observed during recent years [3], leading to the hedgehog being listed as “near threatened” in the IUCN Red List since 2024 [4]. Reasons for this decline are habitat loss and fragmentation, lack of food due to declining insect mass [5], injuries caused by traffic and gardening tools, and infectious diseases including ecto- and endoparasitism [3]. Being a synanthropic species, the European hedgehog lives in close proximity to humans and their pets, and is often presented at veterinary practices [6]. This emphasizes the need for up-to-date knowledge about parasites infecting hedgehogs, and their zoonotic or spillover potential to pets at the wildlife–domestic animal–human interface in the frame of the One Health concept.

The lungworm *Crenosoma striatum* is considered the most important endoparasite of European hedgehogs [6–8], and lungworms in general are assumed to be one of their main causes of death [6, 9]. Besides the hedgehog specific *C. striatum* [10], a second species, *Capillaria aerophila*, may occur in the respiratory tract of hedgehogs as well as various other mammals, including dogs, cats and in rare cases even humans [11–13]. Moreover, *Eucoleus tenuis* has been recorded in hedgehogs, although not in Germany [14, 15]. As this species has been mentioned as a synonym of *C. aerophila* by Carlson [8], the lungworm species distribution in hedgehogs has not been finally clarified. Whereas *C. striatum* is found in the main and secondary bronchi, *C. aerophila* occurs in the small bronchi and can even be found in the lung parenchyma. Both species can lead to respiratory symptoms including cough, dyspnoea and bronchopneumonia in the case of bacterial involvement. The lifecycle of *C. striatum* has been well studied. Females are ovoviviparous, the eggs or larvae are coughed up and then swallowed, and first stage larvae are excreted via the faeces [6, 9, 16]. The third infective larval stage develops in obligatory intermediate hosts (different snail species, e.g. *Cepea nemoralis*, *Cepea hortensis* and *Arianta arbustorum* [10]), which are prey for hedgehogs [6, 9, 16]. In contrast, the lifecycle of *C. aerophila* has not yet been fully clarified. Female worms shed eggs, which are excreted via the faeces, and might directly infect hedgehogs or earthworms as intermediate or paratenic hosts [6, 9, 17].

*Capillaria* spp. are also found in the gastrointestinal tract of hedgehogs. In addition to *Capillaria erinacei*, which was originally reported to parasitise hedgehogs by Rudolphi [18] and was further described by Skrjabin et al. [19], there may be a second gastrointestinal *Capillaria* species as two further *Capillaria* egg types, besides those of *C. aerophila*, have been found by different authors [6, 9, 16, 20]. Some of these authors refer to the second gastrointestinal species as *Capillaria ovoreticulata* [9, 16, 20], as originally described by Laubmeier [16], based on the morphology of eggs and adult worms. The situation is further complicated by the fact that *C. erinacei* has sometimes been regarded as a synonym of *Capillaria putorii*, a species known to parasitise mustelids and felids, among others [21]. Taxonomic controversy also exists around the genus classifications within the family Capillariidae, with frequent reassignment of species. Recent phylogenetic analyses based on the mitochondrial genome placed *C. putorii* within the genus *Aonchotheca* [22]. These hairworm species are present in the gastrointestinal tract and can lead to diarrhoea, dehydration, weight loss and possibly the death of the host [6]. The lifecycle is similar to that of *C. aerophila* with a possible direct transmission or the use of an intermediate or paratenic host [9, 19]. For *C. erinacei*, Romashov [23] proposed that earthworms (*Eisenia rosea*, *Lumbricus terrestris*) may be obligatory intermediate hosts, as uptake of larvated eggs did not result in infection of hedgehogs, whereas feeding of infected earthworms or larvae obtained from earthworms did. However, the experiment relied on a small sample size of only three hedgehogs and remains to be confirmed by further studies.

A less frequently occurring gastric nematode of hedgehogs is the spirurid *Physaloptera clausa* [9]. Further hedgehog specific endoparasites are the trematode *Brachylaemus erinacei*, which settles mainly the intestinal tract but can also be found in the bile ducts [7], and the cestode *Hymenolepis erinacei*, which parasitises in the small intestine [6]. Moreover, acanthocephalan parasites have been found in European hedgehogs [24, 25]. All of these parasites are transmitted via intermediate hosts, namely arthropods in the case of *P. clausa* and *H. erinacei*, snails in the case of *B. erinacei*, and crustaceans or insects in the case of acanthocephalan parasites.

Regarding protozoa, different coccidian species can infect hedgehogs, though *Isospora rastegaievae* seems to be the most important [7, 9]. Furthermore, *Cryptosporidium* spp. and *Giardia* spp., including zoonotic variants (*C. hominis* Ib, *C. parvum* IIa, IIc and IId, and *Giardia duodenalis* assemblage A), have been detected in European hedgehogs [26–28]. These parasites can lead to diarrhoea, dehydration and death of the host. Infection occurs by oral uptake of sporulated oocysts in the case of coccidia and *Cryptosporidium* spp. or cysts in the case of *Giardia* species. Oocysts and cysts are shed by the host, show a high tenacity in the environment and frequently cause reinfections, which constitutes a problem especially in hedgehog stations [9]. For *Cryptosporidium* spp., autoinfection via the thin-walled oocysts is also possible [29].

The aim of the present study was to examine the endoparasite status of European hedgehogs presented at German wildlife rehabilitation centres, and to identify seasonal and host-related infection patterns. To resolve the confusion around the gastrointestinal *Capillaria* spp. of hedgehogs, molecular typing of adult worms was conducted. Furthermore, the species and subgenotype/assemblage distribution of *Cryptosporidium* spp. and *Giardia* spp. was determined to assess their zoonotic potential.

## Methods

### Study animals

European hedgehogs were sampled at three rehabilitation centres located in the northern German federal state of Lower Saxony over a period of 3 years from July 2018 until May 2021, excluding December 2020 and January 2021 [30]. These rehabilitation centres take in weak, sick or injured hedgehogs which are submitted by citizens for further caretaking. During the initial examination, hedgehog data, including their age class, sex and weight, were noted. The age class determination was performed as previously described by Schütte et al. [30], categorizing the animals as juvenile (dependent on mother), subadult (approx. < 1 year) or adult (approx. > 1 year). When rehabilitation was successful, hedgehogs were reintroduced into the wild, whereas moribund animals or those that would not be able to survive in the wild were euthanised or died during the rehabilitation process. Post-mortem examinations were sporadically performed on these animals.

### Sample collection and faecal examination

A single faecal sample of each hedgehog was collected from the animal’s enclosure during routine cleaning procedures without disturbing the animal. Only hedgehogs which were not treated against endoparasites prior to sampling were included. In case the hedgehog died prior to sample collection, a faecal sample was taken directly from the rectum. Samples were stored at 4 °C until examination.

For copromicroscopical examinations, the sample was homogenised using a pestle. A combined sedimentation–flotation method was carried out using an average of 2.0 g (0.1–3.0 g) of faeces. After a sedimentation of 30 min with tap water, 1.5 ml of the sediment was subjected to flotation with 13.5 ml zinc sulphate solution (ZnSO_4_, specific gravity: 1.3), facilitated by centrifugation at 1500 × *g* for 5 min. The entire liquid surface was transferred to a microscope slide and examined for parasite eggs, oocysts and cysts. The entire remaining sediment was subjected to two further sedimentation steps of 3 min each and subsequently stained with one drop of methylene blue (Merck KGaA, Darmstadt, Germany) to detect trematode eggs [31].

For detection of lungworm larvae, the Baermann technique [32] was performed using on average 2.0 g (0.1–3.3 g) of faeces, and larvae were allowed to migrate overnight.

Eggs, oocysts and larvae were differentiated morphologically and counted [20, 33, 34]. The number of parasite stages detected was normalised to 1 g faeces to allow comparisons between the samples.

### Morphological and molecular identification of gastrointestinal *Capillaria* spp. and molecular identification of Acanthocephala spp.

Of two deceased hedgehogs, adult *Capillaria* spp. (six males and six females) were collected from the gastrointestinal tract. These male and female adults as well as intrauterine eggs in the females were compared with previously published morphological descriptions [16, 19, 21, 35]. Furthermore, 33 Acanthocephala spp. were retrieved from the peritoneal cavity of 12 deceased hedgehogs, and a further 10 specimens from the faeces of 5 surviving and 3 deceased hedgehogs.

For *Capillaria* spp. identification, all 12 specimens, and for Acanthocephala, 2 specimens each from the peritoneal cavity and the faeces were subjected to molecular analysis. DNA was isolated using the NucleoSpin Tissue kit (Macherey Nagel, Dueren, Germany) according to the manufacturer’s instructions, with prior disruption by bead-beating (Precellys® 24, Bertin, Montigny-le-Bretonneux, France) at 6500 rpm for 2 × 60 s and incubation in lysis buffer supplemented with proteinase K at 56 °C overnight.

For *Capillaria* species identification, a 400 bp fragment of the mitochondrial cytochrome c oxidase subunit I (*cox-1*) gene was amplified using primers COIFmod and COIRmod [36]. The reaction was carried out in a final volume of 50 μl containing 1.25 U (0.25 μl) DreamTaq polymerase, 5.0 μl 10× DreamTaq buffer (including 20 mM MgCl_2_; Thermo Fisher Scientific, Waltham, MA, USA), 1.0 μl PCR nucleotide mix (0.2 mM each; Roti®-Mix PCR 3; Carl Roth GmbH + Co. KG, Karlsruhe, Germany), 5.0 μl of each primer (1 μM final concentration) and 5 µl template DNA. For amplification, an initial denaturation step at 95 °C for 3 min was followed by 40 cycles of denaturation at 94 °C for 30 s, annealing at 54 °C for 1 min, and extension at 72 °C for 1 min; a final extension was performed at 72 °C for 10 min. In case of multiple bands visible on a GelRed®-supplemented (1:10,000; Biotium, Inc., Fremont, CA, USA) 1.5% agarose gel, the 400 bp band was excised from the gel and centrifuged at 2300 × *g* for 2 min through a pipette filter tip, followed by reamplification using the same PCR protocol and 1 µl filtrate as template.

In case of Acanthocephala spp., a 710 bp *cox-1* sequence was amplified using the primer pair LCO1490 and HCO2198 [37]. The reaction was carried out in a final volume of 50 μl containing 5 U (1.0 μl) DreamTaq polymerase, 5.0 μl 10×  DreamTaq buffer (including 20 mM MgCl_2_; Thermo Fisher Scientific, Waltham, MA, USA), 1.0 μl PCR nucleotide mix (0.2 mM each; Roti®-Mix PCR 3; Carl Roth GmbH + Co. KG, Karlsruhe, Germany), 1.0 μl of each primer (1 μM final concentration) and 5 µl template DNA. For amplification, an initial denaturation step at 95 °C for 3 min was followed by 35 cycles of denaturation at 95 °C for 30 s, annealing at 40 °C for 1 min and extension at 72 °C for 1 min; a final extension was performed at 72 °C for 5 min.

PCR products were Sanger sequenced at Microsynth Seqlab Laboratories (Göttingen, Germany). Obtained sequences were edited in Clone Manager 9 Professional Edition (Scientific & Educational Software, Colorado, USA) and subsequently blasted against the NCBI database. To check intra- and interspecific variation in *Capillaria* spp., the sequences were trimmed to a uniform length of 298 bp and aligned. Obtained sequences were deposited in GenBank under accession numbers PV066222–PV066232 (*Capillaria* spp.) and PV065738–PV065739 (*Plagiorhynchus cylindraceus*).

### FASTest® CRYPTO-GIARDIA

For detection of *Cryptosporidium* spp. and/or *Giardia* spp. antigens, the rapid immunochromatographic *FAST*est® CRYPTO-GIARDIA Strip (MEGACOR Diagnostik GmbH, Hörbranz, Austria) was used. The test was performed according to the manufacturer’s instructions. Depending on the consistency of the faecal sample, one spoon was used for solid–pasty faeces, two spoons for pasty–fluid faeces and three spoons for fluid samples. In five cases with a low amount of available faeces, half a spoon had to be used.

### Molecular identification of *Cryptosporidium* spp. and *Giardia* spp.

DNA of *Cryptosporidium*- and/or *Giardia*-positive samples was isolated using the GeneMATRIX® Stool DNA Purification Kit (Roboklon GmbH, Berlin, Germany) according to the manufacturer´s instructions. Whenever possible, 200 mg of faeces were used, whereas in 13 cases, only 10–170 mg faeces was available.

For *Cryptosporidium* species differentiation, a 900 bp fragment of the 60 kDa glycoprotein gene was amplified using primers AL3531 and AL3535 [38]. If no or only a weak amplicon was achieved, a nested PCR targeting a 400–600 bp fragment of the 60 kDa glycoprotein gene was performed by using primers AL3532 and AL3533 [39]. The reactions were carried out in a final reaction volume of 25 μl containing 1 U (0.2 μl) DreamTaq polymerase, 2.5 μl 10× DreamTaq buffer (including 20 mM MgCl_2_; Thermo Fisher Scientific, Waltham, MA, USA), 0.5 μl PCR nucleotide mix (0.2 mM each; Roti®-Mix PCR 3; Carl Roth GmbH + Co. KG, Karlsruhe, Germany), 0.5 μl of each primer (0.2 μM final concentration) and 5 µl (1st PCR) or 1 µl (2nd PCR) template DNA. For amplification, an initial denaturation step at 95 °C for 3 min was followed by 35 cycles of denaturation at 95 °C for 30 s, annealing at 50 °C for 30 s and extension at 72 °C for 1 min; a final extension was performed at 72 °C for 10 min.

For *Giardia* species differentiation, a 753 bp fragment of the β-giardin gene was amplified using primers G7 and G759 [40]. In the case of no or only a weak amplicon, this was followed by a nested PCR targeting a 511 bp fragment by using primers published by Lalle et al. [41]. The reactions were carried out in a final reaction volume of 25 μl containing 2.5 U (0.5 μl) DreamTaq polymerase, 2.5 μl 10× DreamTaq buffer (including 20 mM MgCl_2_; Thermo Fisher Scientific, Waltham, MA, USA), 0.5 μl PCR nucleotide mix (0.2 mM each; Roti®-Mix PCR 3; Carl Roth GmbH + Co. KG, Karlsruhe, Germany), 0.5 μl of each primer (0.2 μM final concentration) and 5 µl (1st PCR) or 1 µl (2nd PCR) template DNA. For amplification, an initial denaturation step at 95 °C for 3 min was followed by 40 (1st PCR) or 35 (2nd PCR) cycles of denaturation at 95 °C for 30 s, annealing at 65 °C for 30 s and extension at 72 °C for 1 min; a final extension was performed at 72 °C for 7 min. Positive and negative controls were included in all PCR reactions.

The amplicons were visualised on a GelRed®-supplemented (1:10,000; Biotium, Inc., Fremont, CA, USA) 1.5% agarose gel. Sanger sequencing was carried out at the Microsynth Seqlab Laboratories (Göttingen, Germany), and obtained sequences were edited in Clone Manager 9 Professional Edition (Scientific & Educational Software, Colorado, USA) and blasted against the NCBI database. *Cryptosporidium* spp. subtype sequences were further classified on the basis of the number of TCA (A), TCG (G) and ACATCA (R) repeats as suggested by Sulaiman et al. [42]. In case of *Giardia* spp. subgenotype/assemblage differentiation, sequences were aligned with reference sequences from the NCBI database (GenBank accession nos. X14185, AY072723–AY072729, AY545642–AY545649, AY647264–AY647266, AY653159, EU769221 and MT713330) [40, 41, 43–45]. Obtained sequences were deposited in GenBank under accession numbers PV061399–PV061451 (*Cryptosporidium* spp.) and PV061452–PV061458 (*Giardia* spp.).

### Statistical analysis

Statistical analyses were conducted in R v4.4.0. [46]. Absence/presence data of the most prevalent parasites, i.e. *C. striatum*, *C. aerophila*, *C. erinacei* and *C. putorii*, in relation to sampling month, sampling year, animal sex, age class, bodyweight and the amount of analysed faeces were investigated using binomial generalised linear models (GLMs). Owing to missing data, the models were calculated for a subset of 413 animals. The models were compared with a null model containing only the intercept in a likelihood ratio test (R-function ANOVA, test = ‘chisq’). Multivariable modelling of excretion intensities was attempted using GLMs with negative binomial error distributions, but did not result in models with acceptable distributions of residuals.

Faecal examination methods for the detection of *B. erinacei* were compared via McNemar’s test (R-function mcnemar.test) and coccidian prevalence of adult and subadult animals were compared via Chi-squared test.

## Results

### Animal data

**Fig. 1:**
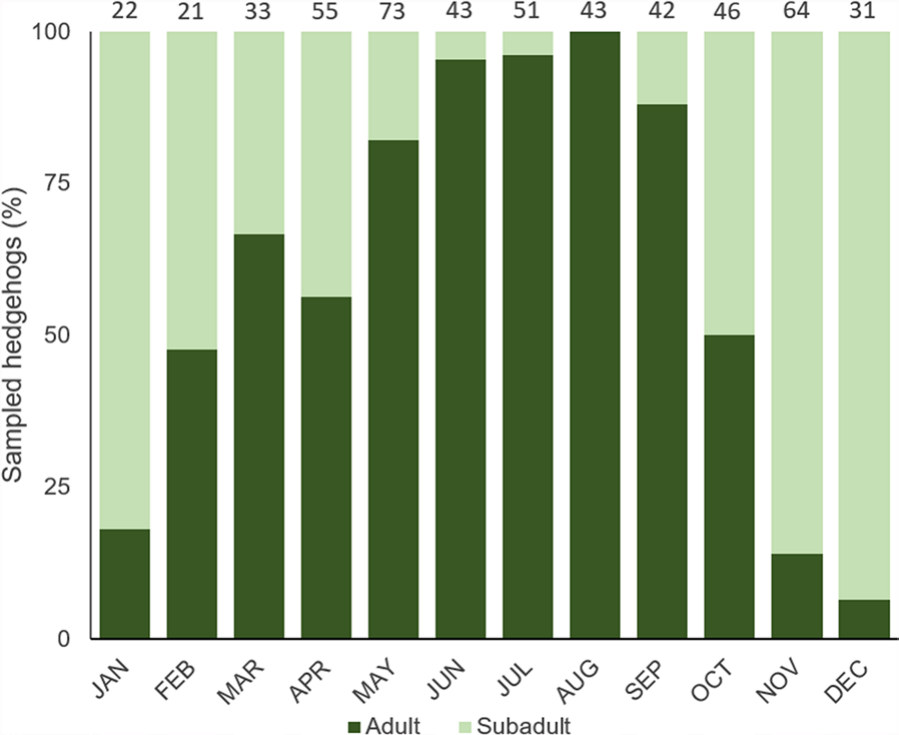
Monthly age class distribution of sampled European hedgehogs. Total numbers of examined animals per month are shown above the bars. The graph includes 524 hedgehogs as the age of seven animals was not determined

Faecal samples of 531 hedgehogs were available for examination. The sex distribution was almost equal, with 47.1% [250/531] males and 47.3% [251/531] females, while the sex was not determined for 5.6% [30/531] of animals. More adult (62.3% [331/531]) than subadult (36.3% [193/531]) hedgehogs were sampled, whereas no juveniles were among the sampled animals, and the age of seven (1.3%) animals was not determined. Adult animals were brought into the rehabilitation centres mainly during the summer months, while subadult animals were taken in more often in autumn and winter (Fig. 1). Regarding the fate of hedgehogs, 62.0% (329/531) could be released back into the wild, 22.8% (121/531) had to be euthanised and 15.1% (80/531) died during the rehabilitation process. The fate of one animal was not recorded.

### Overall endoparasite status

With a frequency of 95.5% (507/531), almost all hedgehogs were positive for at least one endoparasite species. Prevalence values and excretion intensities are summarised in Table 1.

**Table 1:** Prevalence and excretion intensities of endoparasite stages in faecal samples of 531 European hedgehogs (331 adults, 193 subadults, 7 with undetermined age)

Endoparasite	Examination method	No. of infected hedgehogs (%)	No. of infected adult hedgehogs (%)	No. of infected subadult hedgehogs (%)	No. of infected hedgehogs age n.d. (%)	Excretion intensity, FLC/FEC^1^	Excretion intensity, mean	Excretion intensity, median
*Crenosoma striatum*	Baermann technique	405 (76.3)	239 (72.2)	161 (83.4)	5 (71.4)	1–26.623 (n.d. in four samples)^3^	400.7	55
	Flotation^2^	224 (42.2)	112 (33.8)	108 (56.0)	4 (57.1)	1–182	4.4	0.0
	Sedimentation	85 (16.0)	43 (13.0)	40 (20.7)	2 (28.6)	1–87	0.7	0.0
	Total	412 (77.6)	245 (74.0)	162 (83.9)	5 (71.4)	–	–	–
*Capillaria aerophila*	Flotation^2^	142 (26.7)	52 (15.7)	87 (45.1)	3 (42.9)	1–35	1.1	0.0
*Capillaria erinacei*	Flotation^2^	362 (68.2)	192 (58.0)	166 (86.0)	4 (57.1)	1–1210	24.5	2.0
*Capillaria putorii*	Flotation^2^	362 (68.2)	216 (65.3)	141 (73.1)	5 (71.4)	1–194	8.6	2.0
*Brachylaemus erinacei*	Flotation^2^	26 (4.9)	14 (4.2)	11 (5.7)	1 (14.3)	1–158	0.5	0.0
	Sedimentation	7 (1.3)	5 (1.5)	2 (1.0)	0 (0.0)	2–3090	6.0	0.0
	Total	27 (5.1)	15 (4.5)	11 (5.7)	1 (14.3)	–	–	–
Large trematode eggs	Sedimentation	1 (0.2)	1 (0.3)	0 (0.0)	0 (0.0)	2	0.0	0.0
*Hymenolepis nana*	Flotation^2^	1 (0.2)	0 (0)	1 (0.5)	0 (0.0)	1	0.0	0.0
Acanthocephala spp.	Macroscopy	8 (1.5)	6 (1.8)	2 (1.0)	0 (0.0)	1–2	0.0	0.0
Coccidia	Flotation^2^	68 (12.8)	48 (14.5)	16 (8.3)	4 (57.1)	1–3216	9.5	0.0
*Giardia* spp.	Flotation^2^	5 (0.9)	0 (0.0)	5 (2.6)	0 (0.0)	12–1198 (n.d. in two samples)^3^	2.3	0.0
	FASTest® (Megacor)	7 (1.3)	1 (0.3)	6 (3.1)	0 (0.0)	–	–	–
*Cryptosporidium* spp.	FASTest® (Megacor)	63 (11.9)	21 (6.3)	41 (21.2)	1 (14.3)	–	–	–

^1^ *FLC* faecal larval count calculated for 1 g faeces, *FEC* faecal egg count calculated for 1 g faeces

^2^ Combined sedimentation–flotation method

^3^ The excretion intensity of *C. striatum* and *Giardia* spp. was too high to be counted in four and two samples, respectively

*n.d.* not determined

### Pulmonary parasites

**Fig. 2:**
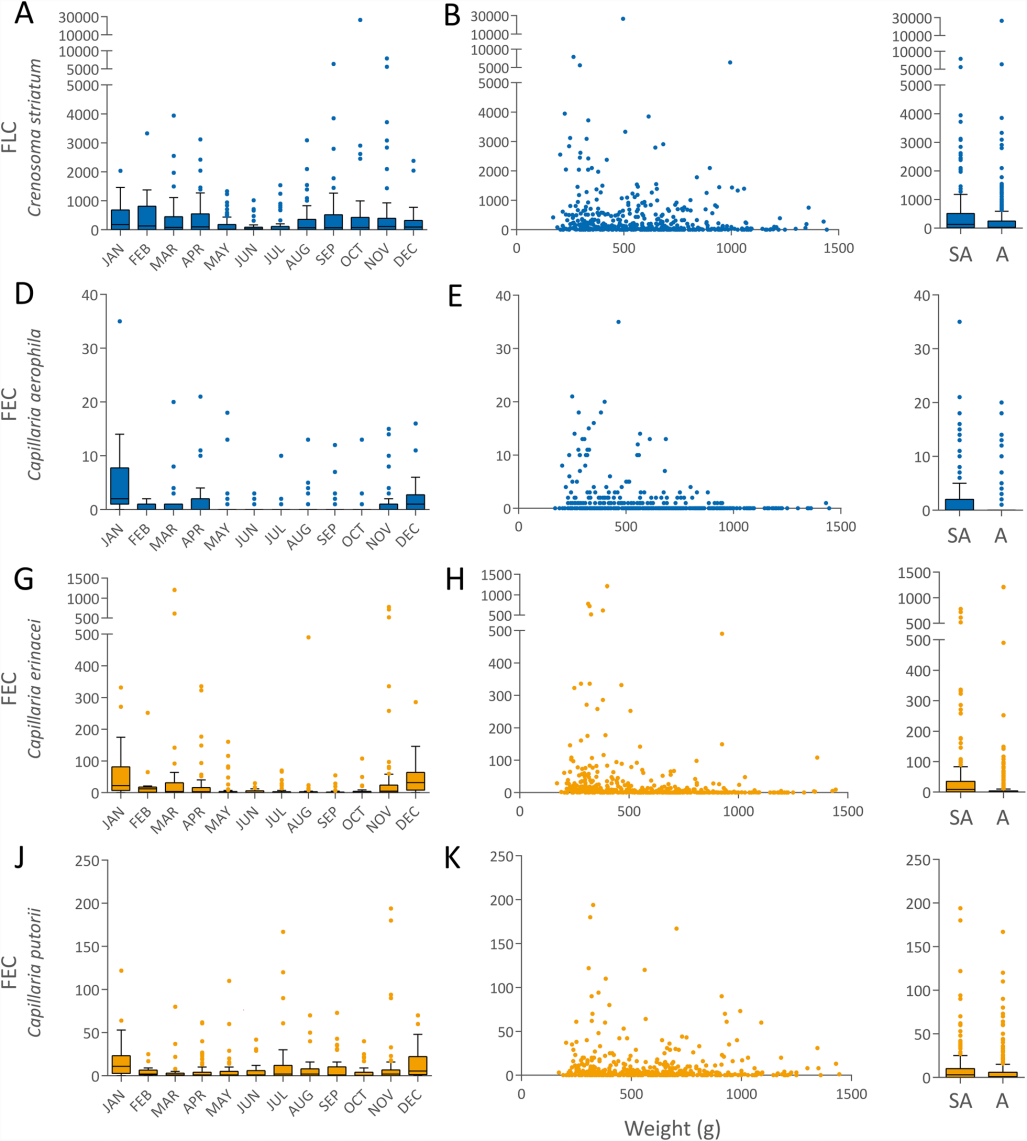
Excretion intensities of *Crenosoma striatum* as determined by the Baermann technique (**A**–**C**) and of *Capillaria aerophila* (**D**–**F**), *Capillaria erinacei* (**G**–**I**) and *Capillaria putorii* (**J**–**L**) as determined by combined sedimentation–flotation in faecal samples of European hedgehogs per month (left), according to hedgehog bodyweight (g; middle) or age class (SA = subadult, A = adult; right). *FLC* faecal larval count calculated for 1 g faeces, *FEC* faecal egg count calculated for 1 g faeces

The most frequently detected endoparasite was *C. striatum* with a prevalence of 77.6% [412/531]. Larvae of *C. striatum* were primarily identified by the Baermann technique, but also by the applied flotation and sedimentation technique. However, the zinc sulphate solution used for flotation can change the morphology of larvae, which is why they could not always be reliably differentiated as *Crenosoma striatum*. No significant association of sampling month and year, sex, age class, bodyweight and the amount of faeces with the probability of *C. striatum* infection was identified, as the GLM was not significantly different from the null model (Table 2). Regarding the excretion intensity, as determined by the Baermann technique, *C. striatum* larval counts appeared to be lower during May, June and July than in the remaining months, and higher in animals with a lower bodyweight (Fig. 2). However, this could not be confirmed statistically as respective multivariable modelling attempts did not result in acceptable models.

**Table 2:** Results of the binomial GLMs testing the influence of seasonal and host-related factors on the infection rate of European hedgehogs with the pulmonary parasites *Crenosoma striatum* (A) and *Capillaria aerophila* (B)

	Model A: *Crenosoma striatum*				Model B: *Capillaria aerophila*			
	Est	SE	*z*	*P*	Est	SE	*z*	*P*
Intercept	1.72	0.92	1.87	0.062	1.76	1.03	1.71	0.088
Sampling month (ref.: January)								
February	0.28	0.93	0.30	0.766	−1.62	0.85	−1.90	0.057
March	0.07	0.86	0.08	0.934	−2.23	0.84	−2.66	**0.008**
April	0.21	0.79	0.26	0.792	−2.15	0.77	−2.79	**0.005**
May	−0.19	0.75	−0.25	0.803	−2.36	0.77	−3.06	**0.002**
June	−0.47	0.79	−0.60	0.548	−1.26	0.80	−1.59	0.112
July	−0.12	0.79	−0.16	0.877	−2.48	0.89	−2.80	**0.005**
August	0.30	0.85	0.35	0.727	−1.57	0.87	−1.80	0.073
September	−0.19	0.80	−0.24	0.808	−1.99	0.84	−2.37	**0.018**
October	−0.39	0.77	−0.51	0.610	−3.48	0.92	−3.78	**< 0.001**
November	0.04	0.79	0.05	0.962	−2.85	0.82	−3.48	**0.001**
December	0.79	1.01	0.78	0.434	−0.49	0.88	−0.56	0.575
Sampling year (ref.: 2018)								
2019	0.39	0.44	0.90	0.370	0.07	0.50	0.13	0.894
2020	0.24	0.43	0.54	0.587	−0.31	0.53	−0.58	0.559
2021	0.51	0.66	0.77	0.439	−0.15	0.69	−0.22	0.823
Sex (ref.: male)	−0.37	0.25	−1.51	0.131	0.19	0.27	0.69	0.490
Age class (ref.: subadult)	0.09	0.43	0.20	0.844	−0.46	0.46	−0.99	0.325
Bodyweight	−0.001	0.001	−2.10	**0.036**	−0.002	0.00	−1.98	**0.048**
Outcome (ref.: released)								
Euthanised	0.35	0.33	1.05	0.292	−0.15	0.38	−0.40	0.693
Died	0.18	0.39	0.45	0.651	0.03	0.43	0.08	0.935
Amount of faeces examined	0.11	0.19	0.55	0.580	0.26	0.24	1.06	0.291

Only model B was significantly different from a null model (A: Chi-squared = 19.7, df = 20, *P* = 0.478; B: Chi-squared = 83.9, df = 20, *P* < 0.001). Significant *P*-values are printed in bold

*Est.* estimate, *SE* standard error

**Fig. 3:**
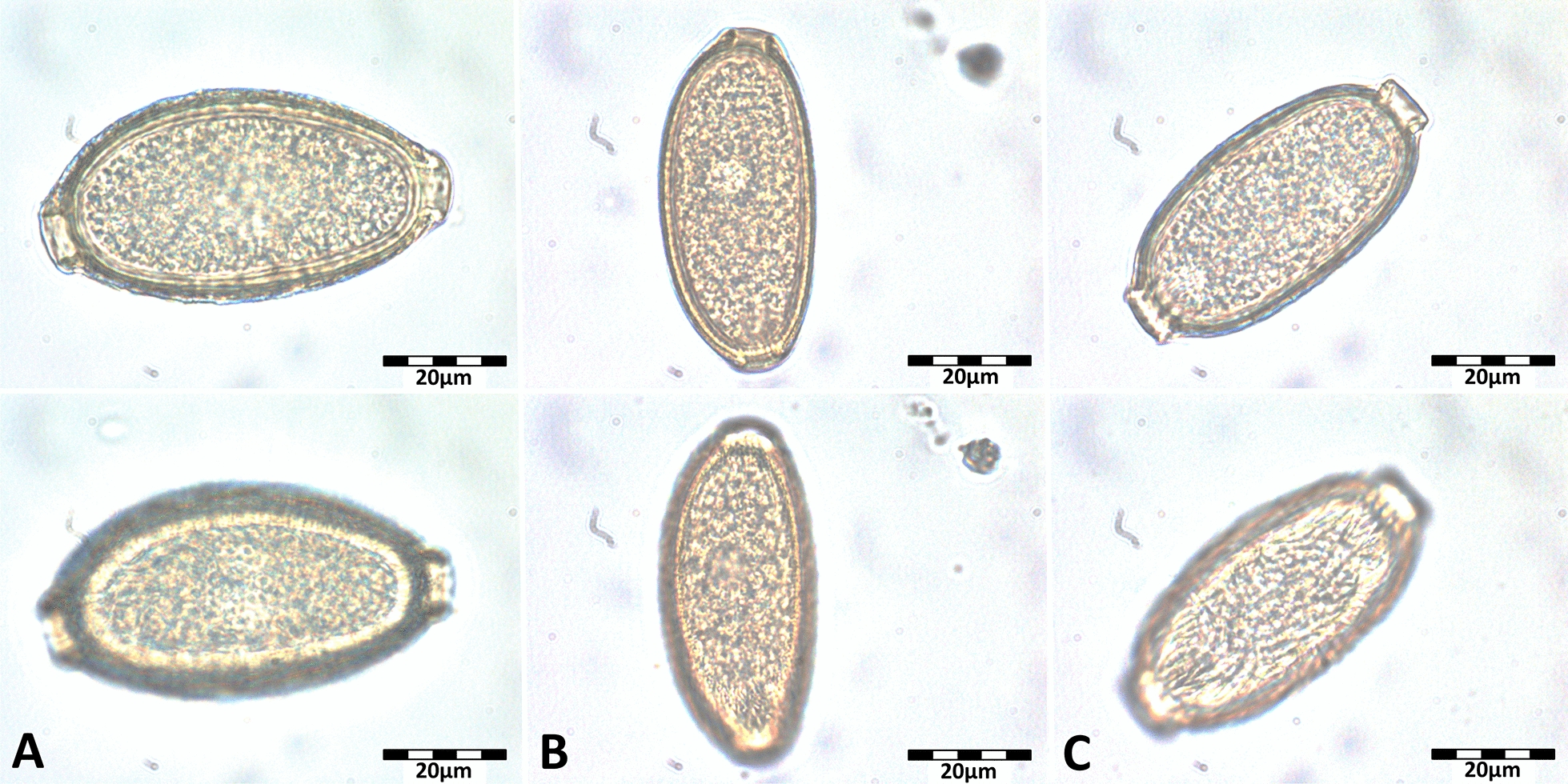
Morphology of *Capillaria* spp. eggs detected in European hedgehog faecal samples. The lower images show the egg surface. *Capillaria aerophila* eggs (**A**) are characterised by a larger size, thick wall and fine-grained surface; *Capillaria erinacei* eggs (**B**) are smaller with a thin wall and fine-grained surface; *Capillaria putorii* eggs (**C**) were of medium size with thin parallel walls and a reticulated surface

**Fig. 4:**
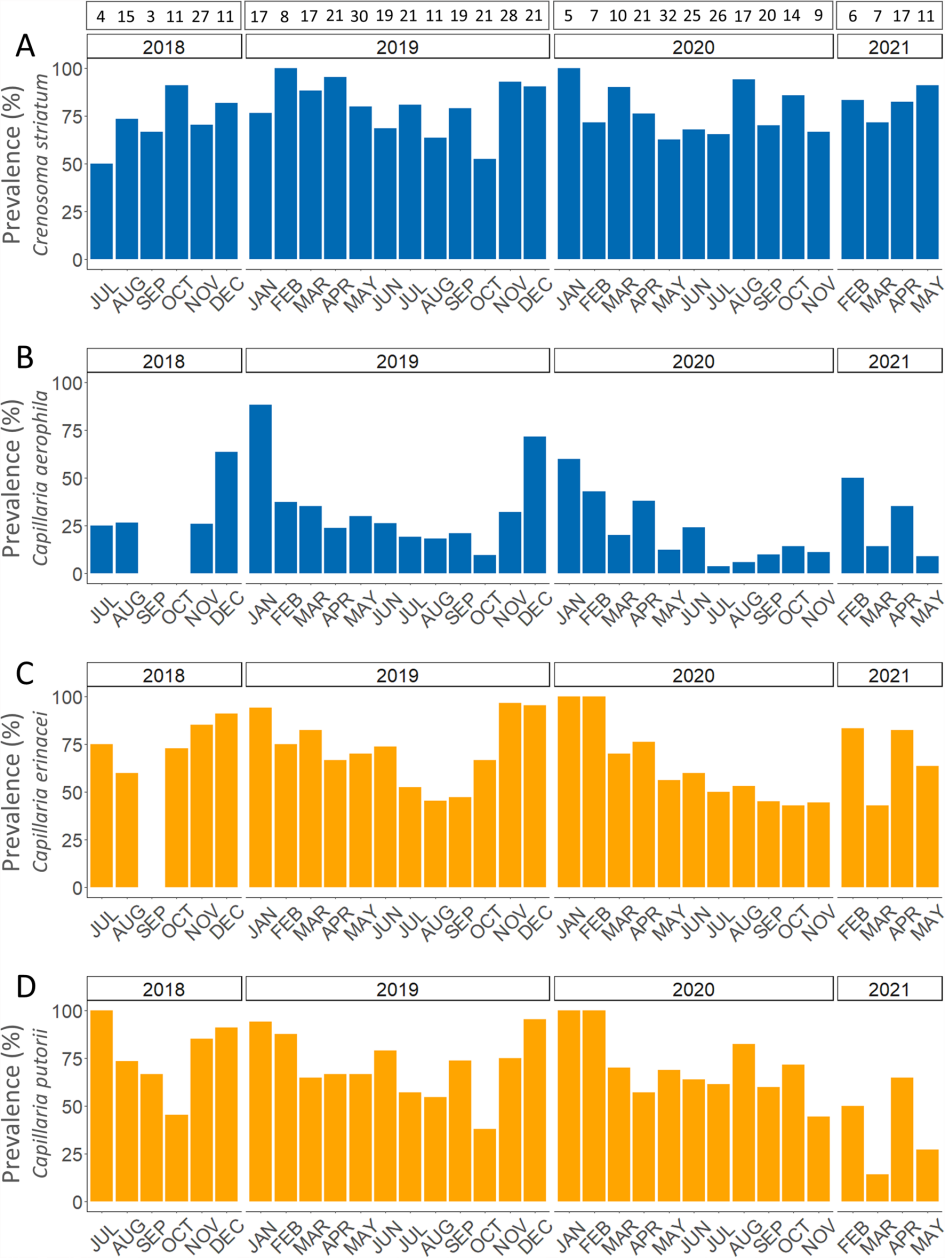
Prevalence of *Crenosoma striatum* (**A**), *Capillaria aerophila* (**B**), *Capillaria erinacei* (**C**) and *Capillaria putorii* (**D**) in faecal samples of 531 European hedgehogs during 2018–2021. Total numbers of hedgehogs examined per month are shown at the top. No animals were examined in December 2020 and January 2021

*Capillaria aerophila* eggs, characterised by their larger size, thick wall and fine-grained surface compared with other capillariid eggs (Fig. 3A), were detected in 26.7% (142/531) of faecal samples. The model calculation for *C. aerophila* showed a significant seasonal influence on the probability of infection, with a lower prevalence from March to May, July, and September to November compared with January (Table 2; Fig. 4). This pattern was more pronounced in adult compared with subadult individuals (Supplementary Fig. S1). Furthermore, animals with a higher bodyweight were significantly less often positive for *C. aerophila* (Table 2). Similar to *C. striatum*, the *C. aerophila* excretion intensity varied throughout the year, and appeared higher in animals with a lower bodyweight (Fig. 2), but again, respective multivariable modelling attempts did not result in acceptable models.

### Gastrointestinal nematodes

**Fig. 5:**
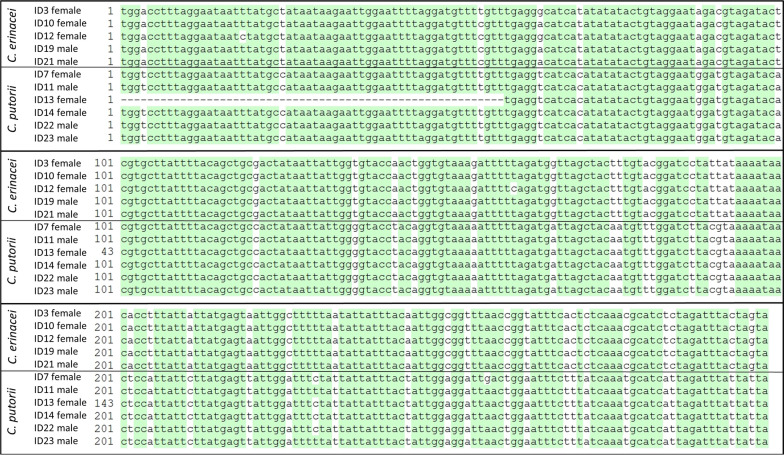
Alignment of *cox-1* sequences (trimmed to 298 bp) obtained from gastrointestinal *Capillaria* species of European hedgehogs

**Fig. 6:**
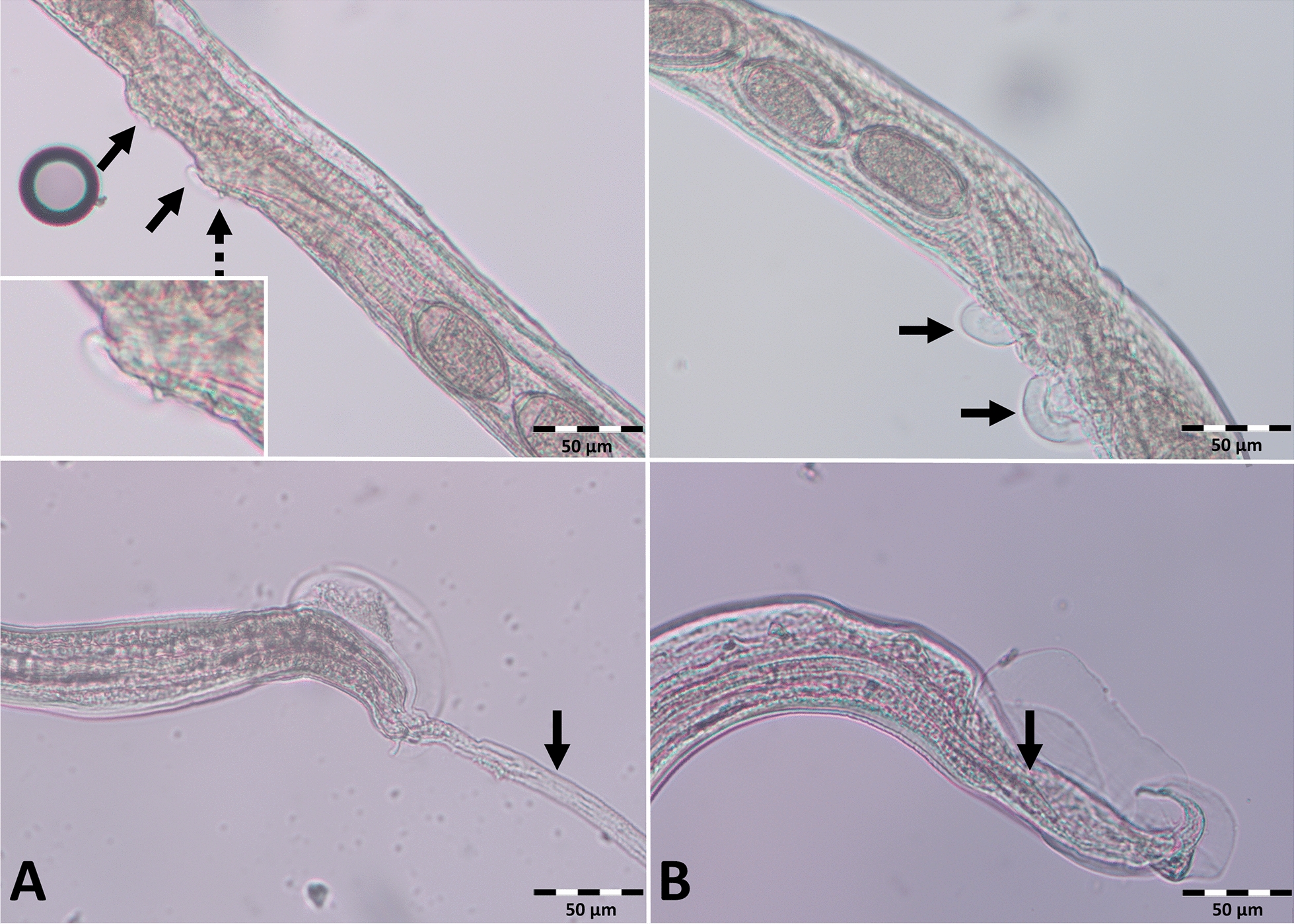
Vulva region of adult female (top) and tail of male (bottom) *Capillaria erinacei* (**A**) and *Capillaria putorii* (**B**) from the gastrointestinal tract of European hedgehogs. The cuticular protrusions of female *C. erinacei* and cuticular expansions of female *C. putorii* as well as the protruded spiculum inside the transversely striated spiculum sheath of *C. erinacei* and the spiculum swelling of *C. putorii* are indicated by black arrows. The dashed arrow indicates the caudal spine at the vulva region of *C. erinacei*, which is enlarged in the insert

Two different egg types of gastrointestinal *Capillaria* spp. were detected. Egg type 1, consistent with *C. erinacei*, was small with a thin wall and a fine-grained surface (Fig. 3B) and occurred in 68.2% (362/531) of faecal samples. The second egg type, which occurred in 68.2% (362/531) of samples, was characterised by parallel walls and a reticulated surface (Fig. 3C). Molecular examination of adult *Capillaria* spp., including females with visible intrauterine eggs, was successful for 11 samples and confirmed the presence of two different species. Three females containing egg type 1, as well as two males, yielded sequences showing 94.4–94.7% identity to a sequence of *C.* (syn. *Aonchotheca*) *erinacei* (accession no. OQ078761; query cover: 100%). Sequences from three females with egg type 2 and three males were 97.9–100% identical to sequences of *C.* (syn. *Aonchotheca*) *putorii* (accession nos. KC355428–30, PP840083–5, MT127001; query cover: 93.0–100%). The trimmed 298 bp sequences from the two species showed only 84.0–86.0% interspecific nucleotide identity, whereas intraspecific identity was 98.0–100% for *C. erinacei* and 99.0–100% for *C. putorii* (Fig. 5). The comparison with morphological adult and egg descriptions confirmed the assignment of the second gastrointestinal *Capillaria* sp. to *C. putorii* (Figs. 3C and 6). Female specimens of *C. putorii* showed cuticular expansions of varying size and number in the vulva region. Males were characterised by a spiculum with a swelling at the proximal end and a pointed tip, two lateral alae and a large subterminal caudal ala connecting two digitiform processes at the posterior end (Fig. 6). Female specimens of *C. erinacei* examined in this study showed cuticular protrusions with a caudal spine in the vulva region. Male specimens were characterised by a transversely striated spiculum sheath as well as two lateral alae and a small subterminal ala at the posterior end (Fig. 6).

For both intestinal *Capillaria* spp., the number of positive samples was significantly higher with an increasing amount of faeces examined (Table 3). No further significant associations were observed for *C. erinacei*, while the GLM indicated a significant decline of the *C. putorii* prevalence in 2021 compared with 2018 (Table 3; Fig. 4; Supplementary Fig. S1).

**Table 3:** Results of binomial GLMs testing the influence of seasonal and host-related factors on the infection rate of European hedgehogs with the gastrointestinal parasites *Capillaria erinacei* (A) and *Capillaria putorii* (B)

	Model A: *Capillaria erinacei*				Model B: *Capillaria putorii*			
	Est	SE	*z*	*P*	Est	SE	*z*	*P*
Intercept	2.82	1.22	2.31	**0.021**	16.03	594.20	0.03	0.978
Sampling month (ref.: January)								
February	−0.83	1.24	−0.67	0.506	−14.76	594.20	−0.03	0.980
March	−1.49	1.15	−1.29	0.197	−16.38	594.20	−0.03	0.978
April	−1.16	1.12	−1.03	0.301	−15.67	594.20	−0.03	0.979
May	−1.47	1.10	−1.33	0.182	−15.95	594.20	−0.03	0.979
June	−1.25	1.13	−1.11	0.269	−14.93	594.20	−0.03	0.980
July	−1.68	1.12	−1.50	0.135	−15.81	594.20	−0.03	0.979
August	−1.57	1.14	−1.37	0.170	−15.60	594.20	−0.03	0.979
September	−2.17	1.13	−1.93	0.054	−15.71	594.20	−0.03	0.979
October	−2.16	1.12	−1.93	0.053	−16.42	594.20	−0.03	0.978
November	−1.12	1.14	−0.98	0.328	−15.35	594.20	−0.03	0.979
December	−0.72	1.30	−0.55	0.581	−14.46	594.20	−0.02	0.981
Sampling year (ref.: 2018)								
2019	−0.20	0.44	−0.45	0.651	−0.59	0.44	−1.35	0.179
2020	−0.17	0.43	−0.39	0.699	−0.46	0.44	−1.05	0.293
2021	−0.09	0.62	−0.14	0.889	−1.91	0.60	−3.18	**0.001**
Sex (ref.: male)	−0.14	0.23	−0.60	0.547	−0.15	0.23	−0.63	0.531
Age class (ref.: subadult)	−0.34	0.41	−0.82	0.414	0.31	0.40	0.77	0.443
Bodyweight	−0.001	0.001	−1.92	0.055	−0.001	0.002	−1.03	0.303
Outcome (ref.: released)								
Euthanised	−0.51	0.30	−1.70	0.089	0.20	0.32	0.62	0.536
Died	−0.05	0.36	−0.15	0.884	0.50	0.37	1.35	0.178
Amount of faeces examined	0.48	0.19	2.56	**0.011**	0.75	0.20	3.80	**< 0.001**

Both models were significantly different from a null model (A: Chi-squared = 66.0, df = 20, *P* < 0.001; B: Chi-squared = 64.6, df = 20, *P* < 0.001). Significant *P*-values are printed in bold

*Est.* estimate, *SE* standard error

Regarding the excretion intensity, no acceptable multivariable models could be calculated for gastrointestinal nematodes. However, the *C. erinacei* egg count appeared to be lower during May to October, and higher in animals with a low bodyweight (Fig. 2). The excretion intensity of *C. putorii* seemed to be more balanced during the year with high numbers in January and December, but no obvious decline during the summer months. Animals with a low bodyweight appeared to excrete higher *C. putorii* egg numbers, but this effect was not as prominent when compared with *C. striatum*, *C. aerophila* and *C. erinacei* (Fig. 2).

### Gastrointestinal trematodes and cestodes

**Fig. 7:**
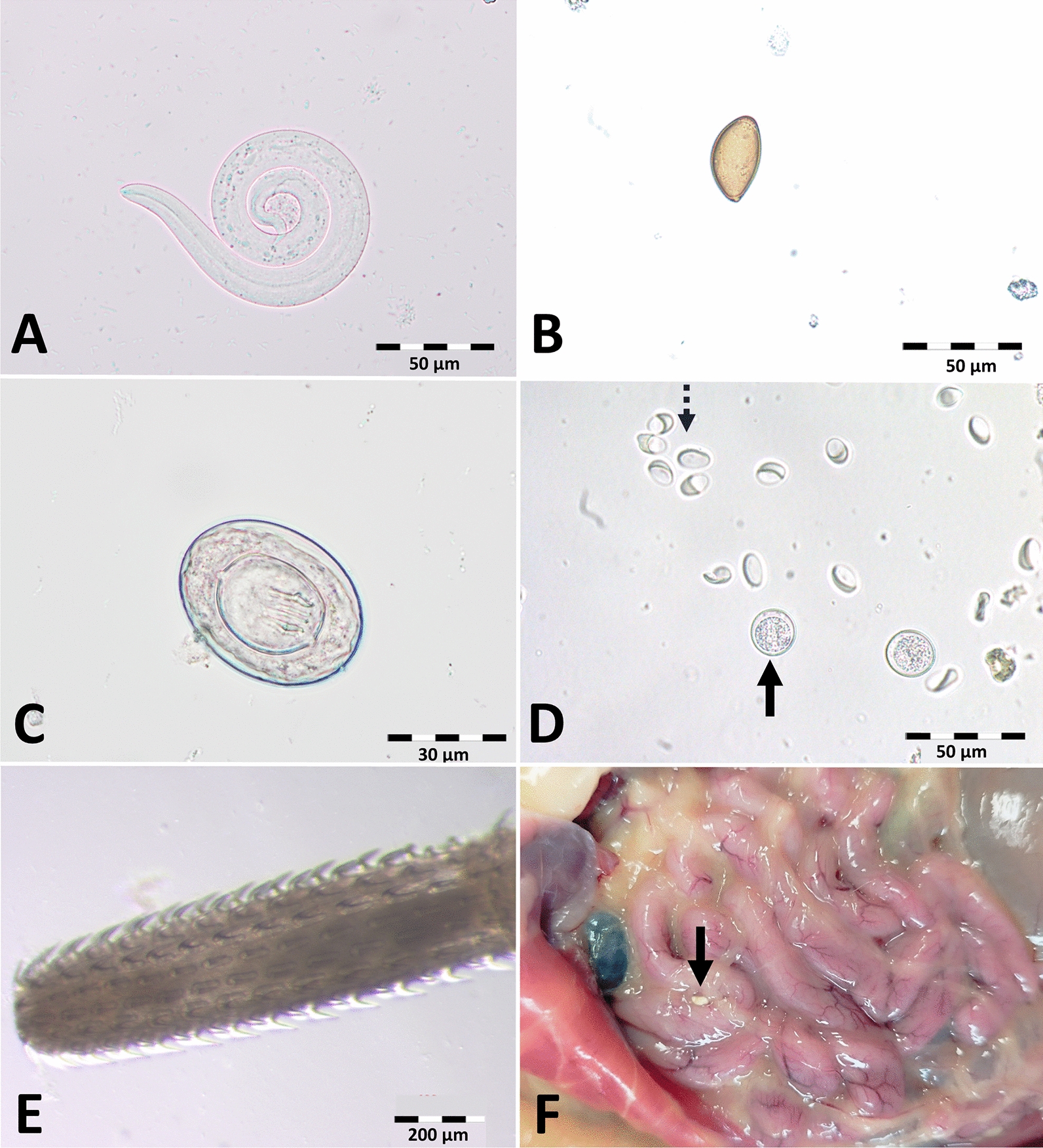
Endoparasites in faecal samples of European hedgehogs. **A** *Crenosoma striatum* larva; **B** *Brachylaemus erinacei* egg; **C** *Hymenolepis nana* egg; **D** coccidian oocysts (solid arrow) and *Giardia* spp. cysts (dashed arrow); **E** proboscis of an acanthocephalan parasite; **F** acanthocephalan parasite (solid arrow) in the abdominal cavity

Eggs of *B. erinacei* were detected in 5.1% (27/531) of hedgehog samples (Table 1; Fig. 7B). Surprisingly, this egg type was detected significantly more often via the flotation (4.9% positive samples) than the sedimentation technique (1.3% positive samples, McNemar test, *χ*^2^ = 15.4, df= 1, *P* < 0.001). Furthermore, in one sample two indeterminable trematode eggs were found (0.2% [1/531]). Regarding cestodes, a single egg was detected in one faecal sample and morphologically determined as *Hymenolepis nana* (0.2% [1/531], Fig. 7C).

### Acanthocephala spp.

In eight (1.5%) faecal samples, up to two specimens of acanthocephalan parasites (Fig. 7E) were detected. As an incidental finding in sporadically dissected hedgehogs, one to six specimens were detected in the peritoneal cavity of 12 animals (Fig. 7F), including one animal whose faecal sample also contained one specimen. Molecular analysis confirmed the species *Plagiorhynchus cylindraceus* of each specimen from the faeces and the peritoneal cavity (98.0–99.5% identity with acc. nos. NC029767, DQ089714, OQ078765, MK300542; query cover: 91.0–100%), while the sequencing of the other two samples was not successful.

### Protozoa

Coccidian oocysts (Fig. 7D) were detected in 12.8% (68/531) of faecal samples. With the prevalence in adult hedgehogs (17.0% [48/331]) twice as high as in subadults (9.0% [16/193]), although statistical significance was not reached (*χ*^2^ = 3.8, df = 1, *P* = 0.0504).

The *FAST*est® CRYPTO-GIARDIA Strip indicated a prevalence of 11.9% (63/531) for *Cryptosporidium* spp. and of 1.3% (7/531) for *Giardia* spp., while copromicroscopy yielded 0.9% (5/531) positive *Giardia* spp. results.

Molecular analysis revealed that six *Giardia*-positive samples contained *G. duodenalis* subassemblage A1, while one sample could only be identified as assemblage A due to low sequence quality. Molecular differentiation was successful for 51 *Cryptosporidium*-positive samples and indicated the presence of *C. parvum* subtype IIa (22% of differentiated samples [11/51]), IIc (10% [5/51]), IId (6% [3/51]) and *C. erinacei* subtype XIIIa (65% [33/51]) and XIIIb (2% [1/51]). In two hedgehogs, coinfections with *C. erinacei* and *C. parvum* were detected, as both species yielded amplicons of different sizes, which were extracted from the agarose gel and sequenced separately. Obtained sequences of *Cryptosporidium* subgenotypes detected in more than one faecal sample were aligned to check percentage identity. Detailed results of the *Cryptosporidium* subgenotype differentiation are shown in Table 4.

**Table 4:** *Cryptosporidium* spp. subtypes in European hedgehogs based on 60 kDa glycoprotein (GP60) amplicon sequencing

Subtype family (no. of positive samples)	Prevalence in differentiated samples (*n* = 51)	Prevalence in all samples (*n* = 531)	Subgenotype (no. of positive samples)	Identity within subgenotype
*C. parvum* IIa (11)	21.6%	2.1%	A13G1R1 (6) A15G1R1 (4)^a^ A15G2R1 (1)	99% 99% n.a.
*C. parvum* IIc (5)	9.8%	0.9%	A5G3 (5)^b^	99%
*C. parvum* IId (3)	5.9%	0.6%	A14G1 (1) A17G1 (1) A18G1 (1)	n.a. n.a. n.a.
*C. erinacei* XIIIa (33)	64.7%	6.1%	A14R10 (1) A17R12 (1)^1^ A17R14 (1) A18R11 (2) A20R6 (1) A20R9 (3) A20R10 (4) A20R11 (2) A21R11 (4) A22R8 (1) A22R10 (1) A22R11 (1) A22R12 (1) A23R8 (1) A23R10 (4)^2^ A23R11 (2) A23R12 (2) A27R10 (1)	n.a. n.a. n.a. 100% n.a. 99% 99% 99% 99% n.a. n.a. n.a. n.a. n.a. 99% 98% 98% n.a.
*C. erinacei* XIIIb (1)	2.0%	0.2%	A17G1R4 (1)	n.a.

Note that only 51 of the 68 *Cryptosporidium*-positive faecal samples could be successfully differentiated

^1^ One coinfection with *C. erinacei* XIIIa A17R12 and *C. parvum* IIa A15G1R1 was determined

^2^ One coinfection with *C. erinacei* XIIIa A23R10 and *C. parvum* IIc A5G3 was determined

*n.a. * not applicable

Within the subtype family *C. erinacei* XIIIa, 28/34 sequences showed underlying signals starting at the transition from the TCA repeat section to the TCAACA repeat section, which indicates that there is one less TCA in a sequence of a possible different coinfecting subgenotype. This effect was particularly evident when the sequences were reamplified by the nested PCR. The same was observed in one *C. parvum* IIdA18G1 positive sample, but not in the remaining 17 *C. parvum*-positive samples. Chromatograms with underlying signals of the subgenotype *C. erinacei* XIIIa A21R11 (GenBank acc. no. PV061430) and *C. parvum* IIdA18G1 (GenBank acc. no. PV061449) are shown exemplarily in Supplementary Figs. S2 and S3.

## Discussion

### Endoparasite prevalence

Almost all (95.5%) examined hedgehogs were infected with one or more endoparasite species. The high prevalence is in concordance with German studies from 1979 (94.3%) [7] and 1988–1992 (90.63%) [47], but higher compared with more recent studies from Germany in 2003–2012 (57.1%) [48] and England in 2010 (69%) [49]. Keeping in mind that in the present study a single sample was taken from each animal and sometimes only a low amount of faeces was available for examination, the true prevalence might be even higher owing to irregular excretion of parasitic stages or an excretion intensity below the detection limit. For gastrointestinal *Capillaria* spp., the multivariable models confirmed a positive correlation between the amount of faeces and detection frequency, and thus, increased sensitivity.

The most frequently detected endoparasite was *C. striatum* with a prevalence of 77.6%. This value is higher compared with studies undertaken since the turn of the millennium (2002–2021, 26.8–51%) in Germany, England and Denmark [15, 48–50], but similar to findings reported during 1979–1999 (50.5–79.4%) in Germany [7, 16, 47]. In contrast, a necropsy-based study conducted in Germany during the same period (1980–2001) reported a prevalence of only 27.2% [51]. However, in these studies it was not certain whether the hedgehogs had been treated against endoparasites prior to examination, except for the study of Laubmeier [16], who examined faecal samples from wild-caught hedgehogs only. Moreover, the studies’ findings were based on a mixture of pathological and copromicroscopical examinations, and Rasmussen et al. [15] examined primarily road kills, thus having a less preselected population compared with the hedgehogs delivered to rescue stations in the present study.

*Capillaria aerophila* is known to infect many mammalian species including the red fox (*Vulpes vulpes*) and the wildcat (*Felis silvestris*), with a prevalence in Germany of 69.4% [52] and 13.0% [53], respectively. Outside Germany, *C. aerophila* was detected in 67.3% and 80.0% of red foxes in Poland [54] and the Netherlands [55], respectively, while 33.8% of dissected wildcats, as well as 24.2% of wildcat’s faecal samples, in Greece tested positive [56]. In addition, *C. aerophila* can also be detected occasionally in cats and dogs [11, 12]. Human infections have rarely been diagnosed [57], with one case leading to bronchial carcinoma-like lesions, showing that symptoms of human *C. aerophila* infection are not very specific [13] and infections may therefore remain undetected. With approximately every fourth hedgehog being infected with this lungworm species, European hedgehogs can play a role in the distribution of *C. aerophila*.

Including *C. aerophila*, three different *Capillaria* egg morphotypes were detected in the present study. Several species names have been suggested for gastrointestinal *Capillaria* spp. of hedgehogs, which were later sometimes considered as synonyms, particularly *C. erinacei*, *C. putorii* and *Capillaria mustelorum*, leading to confusion in the nomenclature [21, 58, 59]. As a consequence, morphological descriptions of *Capillaria* species from hedgehogs are inconsistent in the literature, with many descriptions of *C. erinacei* matching the morphological characteristics of *C. putorii*, especially regarding the striated surface of the egg shell [19, 23, 58, 59]. Moreover, identification of *Capillaria* species can be difficult due to morphological similarities as well as intraspecific morphological variation in different host species [35]. In the present study, the female specimens molecularly identified as *C. erinacei* exhibited a caudal vulvar spine, which was already noticed by Laubmeier [16]. For the second gastrointestinal *Capillaria* species, Laubmeier [16] noted some morphological differences in adult specimens and proposed the name “*C. ovoreticulata*” based on the shape and surface of the eggs. However, the description of this morphotype, which matches the third capillariid egg type in the present study besides *C. aerophila* and *C. erinacei*, also corresponds to the description of *C. putorii*. Nevertheless, the name “*C. ovoreticulata*” was subsequently used in several studies [e.g., 49, 60–62], while Kirillov et al. [63] doubted the existence of *C. ovoreticulata*, as it had not been confirmed genetically. Consequently, molecular investigation was urgently needed to shed light on the gastrointestinal *Capillaria* spp. of European hedgehogs. Here, the morphotype described as “*C. ovoreticulata*” was molecularly identified as *C. putorii*. Moreover, *C. putorii* was recently molecularly detected in European hedgehogs in China [22]. When comparing the obtained *C. putorii* and *C. erinacei cox-1* sequences with publicly available sequences from GenBank, the latter showed slightly lower intraspecific identities (data not shown) than those of the present study. This could be due to the fact that the GenBank sequences originated from different host species, namely the Algerian hedgehog in the case of *C. erinacei*, and the red fox, wildcat and raccoon in the case of *C. putorii*. Overall, the finding that *C. putorii* occurs in European hedgehogs sheds new light on the lifecycle and epidemiology of this parasite, particularly given the high prevalence of nearly 70%. The involvement of hedgehogs could also facilitate the spread of *C. putorii* among domestic cats, as they share private gardens as a habitat.

For the hedgehog-specific trematode *B. erinacei*, considerable regional prevalence variation has been reported [7]. A German study in 1999 showed 22.3% infected hedgehogs in Berlin compared with only 7.2% in Dresden [47]. In the present study, the prevalence was low, with 5.1% positive samples. However, it should be considered that *B. erinacei* eggs with a length of 30–35 µm are quite small [33] and can more easily be missed [6] than larger eggs. *Brachylaemus erinacei* eggs were detected significantly more often via the flotation than the sedimentation technique. The same is known for the similarly sized eggs of *Dicrocoelium dendriticum*, which are detected more reliably by flotation than sedimentation when a flotation solution with a specific gravity > 1.3 is used [64].

In the sediment of one faecal sample, two large trematode eggs were noted. Trematode species excreting large eggs, such as *Isthmiophora melis* and *Nephrotrema truncatum*, have been previously found in hedgehogs, as summarised by Rasmussen et al. [15]. The trematode eggs detected were approximately 130 µm in length, which corresponds to the size described for *I. melis* (120–140 µm [65]), whereas *N. truncatum* eggs measure only 72–95 µm [66]. Nevertheless, it was not possible to determine the trematode species with certainty owing to the low number of eggs.

The hedgehog-specific cestode *Hymenolepis erinacei* could not be detected in this study, in line with previous investigations reporting low prevalence values ranging from 0.0–3.7% [7, 16, 50, 51, 67]. Overall, only a single cestode egg was detected in one sample, morphologically consistent with *H. nana*. This species infects rodents and is considered the cause of the most common human tapeworm infection worldwide [34, 68, 69]. *Hymenolepis nana* can either be transmitted directly or via insects as intermediate hosts. To the authors’ knowledge, *H. nana* has not been previously reported in European hedgehogs. It remains unclear whether the hedgehog was infected with *H. nana* or whether the egg represented a spurious parasite after ingestion of an infected rodent. Especially when food availability is low, hedgehogs are known to feed on carrion [1]. In any case, the fact that just one hedgehog was positive and only one egg was detected indicates that hedgehogs probably do not contribute to the distribution of *H. nana* and that the zoonotic infection risk when taking care of hedgehogs is negligible.

In 1.5% of examined faecal samples, acanthocephalan parasites were incidentally detected. In addition, these parasites were found in the abdominal cavity of 12 animals. Since no systematic dissection of all deceased or euthanised animals was performed, it can be assumed that the actual prevalence was higher. Two specimens were molecularly identified as *P. cylindraceus,*, which has been found in German European hedgehogs previously [24]. As the specimens found were morphologically similar, it is probable that they all belonged to this species. However, owing to the partly strong encapsulation and the morphological similarity of some species this is not certain. Passerine birds, which include avian scavengers such as corvids, are the definite hosts of *P. cylindraceus* [70, 71], but owls [72] have also been found infested with this species. Furthermore, woodlice are used as intermediate hosts, in which the acanthor develops to cystacanth. Extraintestinal cystacanths of *P. cylindraceus* have been found not only in hedgehogs [24] but also in shrews where the cystacanths show a similar morphology to those found in isopods [73, 74]. While Skuballa et al. [24] proposed that the life cycle of this parasite might include hedgehogs as paratenic hosts, which can become a food source for scavenging birds after their death, Coady et al. [73] suggested that parenteral occurrence of cystacanths in shrews is a historic inheritance from ancestors rather than a selective adaptation of this parasite. However, the possible inclusion of hedgehogs in the life cycle of *P. cylindraceus* needs to be further investigated.

Regarding protozoa, coccidian oocysts (12.8%) and *Cryptosporidium* spp. antigen (11.9%) were detected at a similar frequency, whereas a much lower prevalence was determined for *Giardia* spp. (1.3%). For *Giardia* spp., the *FAST*est® CRYPTO-GIARDIA was more sensitive than the flotation. A similar *Cryptosporidium* spp. detection frequency of 9.0% has been described previously [28], while Dyachenko et al. [75] determined a much higher prevalence of nearly 30% in a European hedgehog population preselected for diarrhoea, weakness or anorexia. Wright [76] described both *Cryptosporidium* and *Giardia* infections as mainly subclinical in hedgehogs, but some *Cryptosporidium* spp. positive animals in the present study showed a severely impaired health status with apathy, anorexia, dehydration, hypothermia and green mucoid to watery diarrhoea. Similar symptoms were reported previously [77], leading to the conclusion that *Cryptosporidium* spp. infections can be asymptomatic in hedgehogs, but may also be life-threatening if left untreated, especially in subadult animals [77], and should be considered as a differential diagnosis in cases of diarrhoea. Studies on *Giardia* spp. in German hedgehogs are scarce, but a previous examination reported a prevalence of 6.3% in faecal samples of rehabilitated hedgehogs [78]. Furthermore, *Giardia* spp. have also been reported in hedgehogs in the Netherlands [28], in France [79] and in New Zealand [80].

### Zoonotic potential of detected *Cryptosporidium* and *Giardia* spp.

Both *Cryptosporidium* and *Giardia* have a broad genetic diversity and host spectrum. Molecular analyses in the present study detected the genotype families *C. erinacei* XIIIa and XIIIb, and *C. parvum* IIa, IIc and IId in European hedgehogs, similar to previous findings [26, 28, 75, 81, 82]. In addition, coinfections with *C. erinacei* XIIIa and *C. parvum* IIa in one sample and *C. erinacei* XIIIa and *C. parvum* IIc in another sample were confirmed. As expected, *C. erinacei* was the most frequently detected species, but identification of the subtype family XIIIb, described in farmed and pet African pygmy hedgehogs [83], was unexpected, and to the authors’ knowledge, this is the first report of this subtype family in a European hedgehog. Besides different genotypes, the present study revealed the occurrence of many subgenotypes, especially in the case of *C. erinacei* XIIIa. It is not yet known exactly what information can be derived from subgenotyping *Cryptosporidium* samples, although an association with virulence has been proposed [84]. It remains unclear whether the underlying chromatogram signals indicating a TCA deletion in nearly all *C. erinacei* positive samples are due to coinfections with different *C. erinacei* subgenotypes or represent a sequencing artefact.

In humans, *C. hominis* and *C. parvum* are responsible for most infections [85, 86], but multiple other species exhibit zoonotic potential, including *C. cuniculus*, *C. meleagridis*, *C. equi*, *C. ubiquitum* and *C. felis* [87], as well as *C. erinacei* [84, 88]. The latter species is considered mainly hedgehog-specific; however, both detected subtype families, *C. erinacei* XIIIa [89] and XIIIb [90], have been implicated in human infections. Both cases occurred in immunocompetent middle-aged persons without previous contact with hedgehogs, and the patients showed symptoms of gastroenteritis.

All *Giardia* positive samples were assigned to *G. duodenalis* subassemblage A1, except for one sample which unfortunately could not be assigned to subassemblage A1 with certainty owing to two base substitutions. Both assemblage A [28] and subassemblage A1 [79] have previously been detected in European hedgehogs. Within assemblage A, subassemblage A2 occurs most frequently in human samples [91]. It has even been proposed that this subassemblage should be elevated to the species level under the name *G. hominis* [92]. In contrast, subassemblage A1 is considered mainly zoonotic, being found in humans and animals [91].

### Seasonal and host-related patterns and survival rates

Significant seasonal endoparasite prevalence differences were only evident for *C. aerophila*, with a lower percentage of egg-excreting hedgehogs during spring, summer and autumn compared with winter. One reason could be that hedgehogs brought to rehabilitation centres in winter, a time when healthy individuals are hibernating, often have an impaired immune system. Regarding excretion intensities, the observed patterns for *C. striatum*, *C. aerophila* and *C. erinacei* point towards higher average larvae or egg counts during the colder months. However, this could not be confirmed statistically by multivariable modelling, as no satisfactory models could be derived. Laubmeier [16] also noticed seasonal changes in the excretion intensity of *C. striatum* larvae and *C. aerophila* eggs, but not for gastrointestinal *Capillaria* spp. eggs. However, in that study wild-caught hedgehogs were examined from May to October, thus the winter months were not covered. The patterns in excretion intensities might be driven by an age influence, as subadult animals, which were predominantly sampled during the colder months, seem to show higher excretion intensities, in addition to the challenging winter conditions mentioned above. The significant difference in *C. putorii* prevalence between the years 2018 and 2021 needs to be treated with caution, as sampling in 2021 took place only during 4 months, so this finding may have been incidental.

The multivariable models indicated no significant association of animal sex or age class with endoparasite prevalence, despite the fact that higher prevalence values were observed in subadult rather than adult hedgehogs throughout the year for all endoparasites except coccidian oocysts. For *C. striatum* and *B. erinacei*, a higher prevalence in subadult rather than adult hedgehogs was also observed in a dissection study from the Czech Republic [25]. Conversely, Mariacher et al. [93] reported a higher prevalence of various endoparasite species in adult than subadult animals, although this was based on only a small number of animals (25 adults, 15 subadults).

It should be kept in mind that a preselected population of animals brought to rehabilitation centres was examined in the present study. Especially subadult animals were often taken in due to general health problems such as anorexia and failure to gain weight, symptoms closely associated with endoparasite infections, whereas causes for intake of adult animals were more diverse, including many traumatic injuries. Especially coccidian infections are considered a problem in juvenile and subadult hedgehogs, probably due to a lack of immunity formation as in domestic animal species [94–97]. Thus, routine anticoccidial as well as other antiparasitic treatments in rehabilitation centres are common. Interestingly, this age predisposition towards subadult hedgehogs was not reflected in the present study, as the prevalence of coccidian oocysts was almost twice as high in adult (17.0%) than in subadult (9.0%) animals. Furthermore, the excretion intensity was comparatively low for both subadult and adult animals, so routine treatment was not deemed necessary in general.

Regarding bodyweight, heavier animals were significantly less often positive for *C. aerophila*, whereas no significant correlation was observed for gastrointestinal *Capillaria* species. Furthermore, egg excretions were often higher in animals with a lower bodyweight. A low bodyweight is often associated with an underlying health problem, but might also be due to direct effects of an endoparasite infection. A previous study by Pfäffle [98] showed that gastrointestinal *Capillaria* spp. may have a negative influence on the body condition of European hedgehogs.

No significant associations between endoparasite infections and the survival rate of the examined hedgehogs were observed. This could be owing to the generally high infection rate, as well as a subsequent treatment in the rehabilitation centres. Furthermore, the clinical problems the hedgehogs were presented with were quite diverse, including traumatic injuries and myiasis as reasons for death or euthanasia, which are independent from endoparasite infections. Thus, the present study could not confirm previous reports of endoparasites as a major cause of death in hedgehogs [7, 99].

## Conclusions

The European hedgehog is frequently infected by different endoparasite species, with lungworms and gastrointestinal *Capillaria* spp. representing the most common ones. Regarding the gastrointestinal *Capillaria* spp., molecular examination showed that in addition to *C. erinacei*, hedgehogs were infected with *C. putorii*, previously described as *C. ovoreticulata* on the basis of egg shell morphology. Although not statistically confirmed in the present study, endoparasites might contribute to hedgehog mortality. Thus, when hedgehogs are admitted to rehabilitation centres or veterinary practices, examination for and treatment against parasites should be a priority. Furthermore, endoparasites with zoonotic potential were detected, posing a risk for pet animals and humans. In particular, the molecular differentiation of *Cryptosporidium* and *Giardia* spp. in the present study revealed subtype families and subassemblages known to affect humans. Although no human cases have been reported with a direct connection to hedgehogs yet, hygiene is of major importance when caring for hedgehogs to prevent cross-species transmission.

## Supplementary Information

Supplementary Material 1. Fig. S1. Prevalence of *Crenosoma striatum* (A), *Capillaria aerophila* (B), *Capillaria erinacei* (C) and *Capillaria putorii* (D) in faecal samples of all 531 hedgehogs examined (left), of 331 adult hedgehogs (middle) and 193 subadult hedgehogs (right) during the years 2018–2021. The numbers of hedgehogs examined per month are shown at the top. No animals were examined in December 2020 and January 2021

Supplementary Material 2. Fig. S2. Exemplary chromatogram with underlying signals of the subgenotype *Cryptosporidium erinacei* XIIIa A21R11

Supplementary Material 3. Fig. S3. Exemplary chromatogram with underlying signals of the subgenotype *Cryptosporidium parvum* IId A18G1

## Data Availability

Data supporting reported results is contained within the article. Generated sequences were deposited at GenBank under accession nos. PV066222–PV066232, PV065738–PV065739, PV061399–PV061451 and PV061452–PV061458.

## Abbreviations

BLAST: Basic local alignment search tool
GLM: Generalised linear model
IUCN: International Union for Conservation of Nature
NCBI: National Centre for Biotechnology Information
