# Supplementary figures and images for: Endoparasites of European hedgehogs (Erinaceus europaeus) in Germany and their zoonotic potential: proposed Capillaria ovoreticulata genetically identified as Capillaria putorii
Source: Parasit Vectors. 2025 Jun 7;18:214. doi: 10.1186/s13071-025-06858-0 (PMC12145657; doi:10.1186/s13071-025-06858-0)

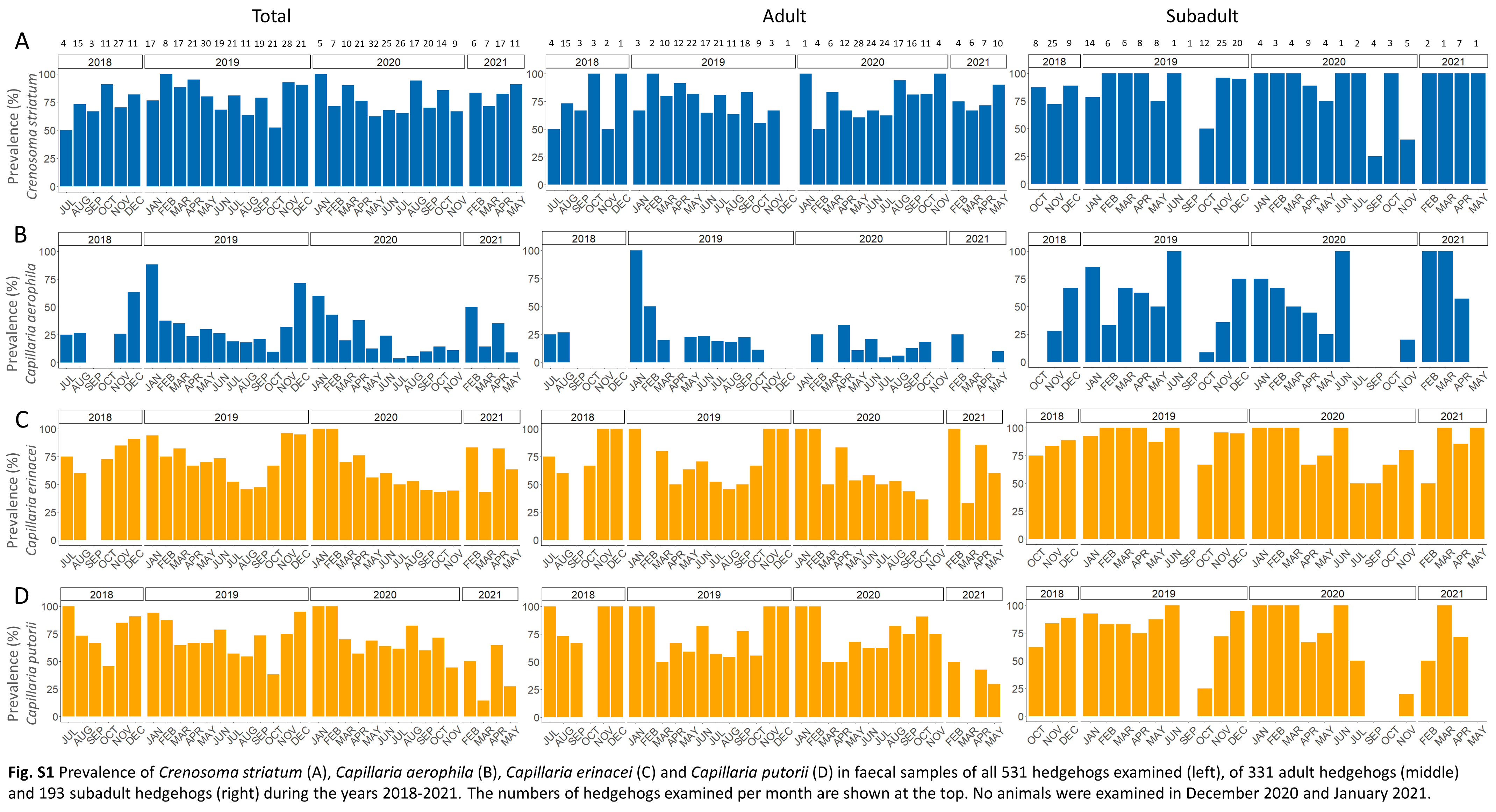

Supplement: Supplementary file 1 — Supplementary Material 1. Fig. S1. Prevalence of Crenosoma striatum (A), Capillaria aerophila (B), Capillaria erinacei (C) and Capillaria putorii (D) in faecal samples of all 531 hedgehogs examined (left), of 331 adult hedgehogs (middle) and 193 subadult hedgehogs (right) during the years 2018–2021. The numbers of hedgehogs examined per month are shown at the top. No animals were examined in December 2020 and January 2021 [file 13071_2025_6858_MOESM1_ESM.png]

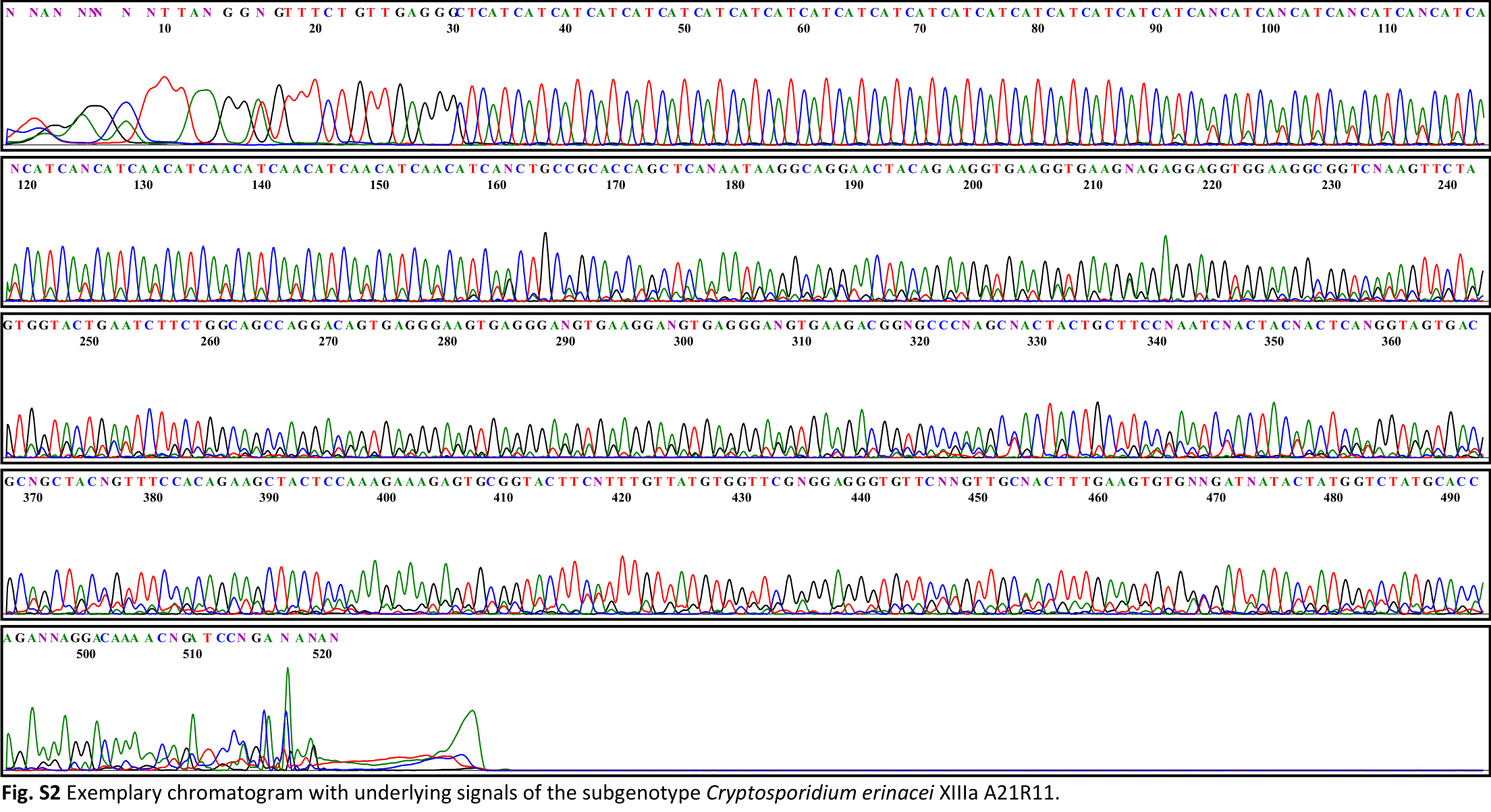

Supplement: Supplementary file 2 — Supplementary Material 2. Fig. S2. Exemplary chromatogram with underlying signals of the subgenotype Cryptosporidium erinacei XIIIa A21R11 [file 13071_2025_6858_MOESM2_ESM.png]

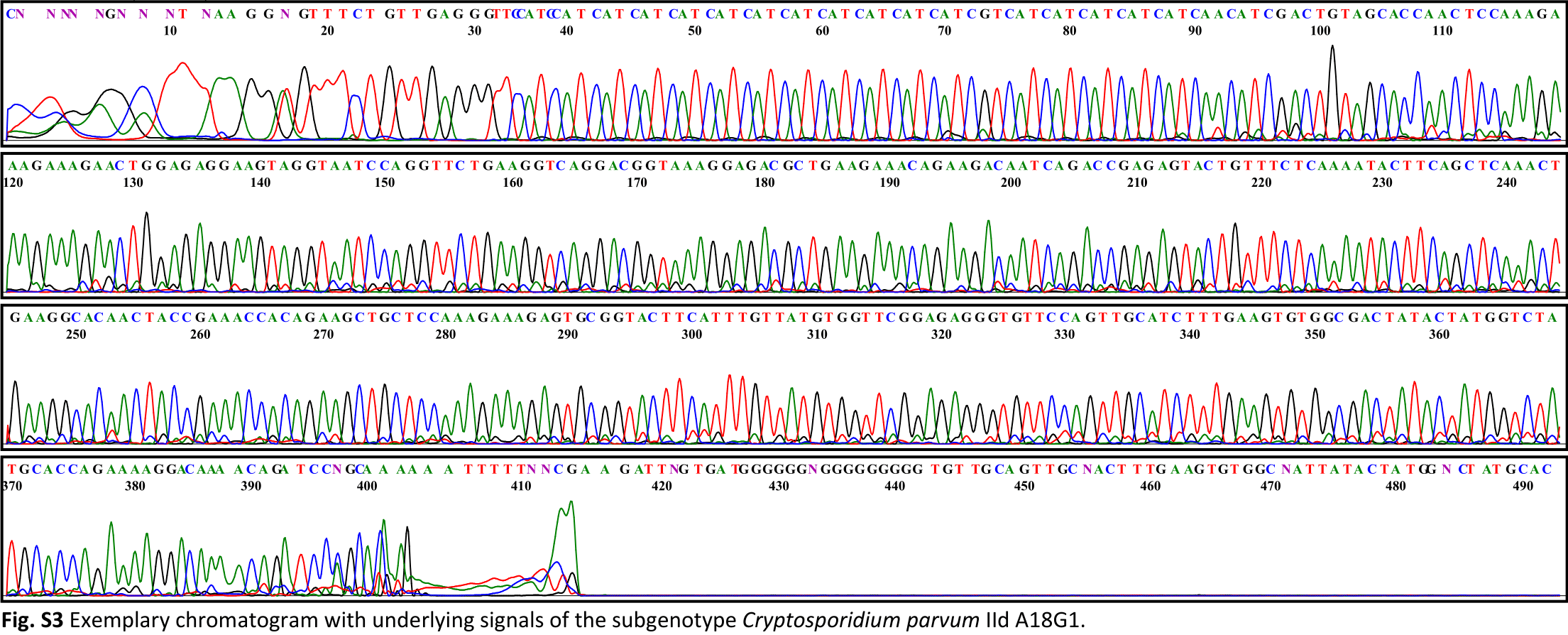

Supplement: Supplementary file 3 — Supplementary Material 3. Fig. S3. Exemplary chromatogram with underlying signals of the subgenotype Cryptosporidium parvum IId A18G1 [file 13071_2025_6858_MOESM3_ESM.png]
